# The genera *Rugonectria* and *Thelonectria* (Hypocreales, Nectriaceae) in China

**DOI:** 10.3897/mycokeys.55.34527

**Published:** 2019-07-01

**Authors:** Zhao-Qing Zeng, Wen-Ying Zhuang

**Affiliations:** 1 State Key Laboratory of Mycology, Institute of Microbiology, Chinese Academy of Sciences, Beijing 100101, China Institute of Microbiology, Chinese Academy of Sciences Beijing China

**Keywords:** Morphology, Multigene analyses, Taxonomy

## Abstract

Recent collections and herbarium specimens of *Rugonectria* and *Thelonectria* from different regions of China were examined. Using combined analyses of morphological and molecular data, 17 species are recognised including three species of *Rugonectria* and 14 species in *Thelonectria*. Amongst them, *R.microconidia* and *T.guangdongensis* are new to science. *Rugonectriamicroconidia* on mossy bark is characterised by superficial, yellow to orange, pyriform to subglobose perithecia with a warted surface; ellipsoidal to broadly ellipsoidal, striate, uniseptate ascospores; and allantoid to rod-shaped, aseptate microconidia. *Thelonectriaguangdongensis* possesses bright red perithecia with a slightly roughened surface and a prominently dark papilla; ellipsoidal, smooth, uniseptate ascospores; and subcylindrical, slightly curved, multiseptate macroconidia. Morphological distinctions and sequence divergences between the new species and their close relatives are discussed. Name changes for the previously recorded species in China are noted.

## Introduction

The family Nectriaceae was introduced in 1865 and circumscribed to accommodate the hypocrealean species having ascomata that are generally yellow, orange-red to purple and usually changing colour in potassium hydroxide (KOH) and lactic acid (LA) (Rossman et al. 1999). About 55 genera containing 900 species are included in the family (Lombard et al. 2015). A phylogenetic backbone for Nectriaceae was constructed based on DNA sequences of 10 loci by Lombard et al. (2015).

The genus *Rugonectria* P. Chaverri & Samuels, typified by *R.rugulosa* (Pat. & Gaillard) Samuels, P. Chaverri & C. Salgado, is characterised by perithecia solitary or in groups, seated on or partially immersed in a stroma. The perithecia are orange to red, globose to subglobose and non-papillate, with warted or rugose walls. Ascospores are ellipsoidal to oblong, striate, hyaline and 1-septate; and microconidia are ovoid to cylindrical (Chaverri et al. 2011). Currently, four species are recognised in the genus (Chaverri et al. 2011; Zeng et al. 2012). *Thelonectria* P. Chaverri & C. Salgado, typified by *T.discophora* (Mont.) P. Chaverri & C. Salgado, was established by Chaverri et al. (2011) to accommodate the nectriaceous fungi having superficial, globose to subglobose or pyriform to elongated perithecia which do not collapse when dry, with a prominent and darkened papilla; smooth, rarely spinulose or striate ascospores and curved macroconidia with rounded ends (Chaverri et al. 2011; Lombard et al. 2015; Salgado-Salazar et al. 2016). About 44 species are currently accepted in the genus (Chaverri et al. 2011; Salgado-Salazar et al. 2012, 2015, 2016; Zeng and Zhuang 2013; Crous et al. 2018). Species in the genera *Rugonectria* and *Thelonectria* are distributed in the tropics, subtropics and temperate regions and occur on early decaying bark, roots, branches, trunks and rarely in soil (Chaverri et al. 2011; Salgado-Salazar et al. 2015). A few species are plant pathogenic, such as *R.castaneicola* (W. Yamam. & Oyasu) Hirooka & P. Chaverri causing *Abies* and *Acer* cankers and *T.rubi* (Osterw.) C. Salgado & P. Chaverri causing *Rubus* cankers (Cedeño et al. 2004; Kobayashi et al. 2005; Chaverri et al. 2011; Salgado-Salazar et al. 2015).

The first record of *Rugonectria* from China dates back to 2000 when *R.rugulosa* (as *Nectriarugulosa* Pat. & Gaillard) was reported by Lu et al. (2000) based on a specimen collected on dead petioles of king palm. Research on *Thelonectria* in China was started by Teng (1936) when *T.discophora* (as *N.discophora* Mont.) was first reported on bark of fallen branches from Yunnan Province. In connection with our current work on the Chinese fungus flora, fresh materials and herbarium specimens of the two genera were examined. Based on morphology and phylogenetic analyses of the partial sequences of α-actin (ACT), internal transcribed spacer (ITS), nuclear ribosomal large subunit (LSU) rDNA and the largest subunit of RNA polymerase II (RPB1), 17 species were identified, including two undescribed species. Morphological and molecular diagnostic features between the new taxa and their closely related fungi are discussed.

## Materials and methods

### Sampling and morphological studies

Specimens were collected from Beijing, Fujian, Guangdong, Hainan, Henan, Hubei, Hunan and Yunnan provinces and are deposited in Herbarium Mycologicum Academiae Sinicae (HMAS) and cultures are kept in the State Key Laboratory of Mycology, Institute of Microbiology, Chinese Academy of Sciences. The methods used by Luo and Zhuang (2010) and Chaverri et al. (2011) were followed for morphological observations. The ascomatal wall reactions to 3% KOH and 100% LA were tested. To observe micromorphological characteristics of perithecial walls, sections were made with a freezing microtome (YD-1508-III, Jinhua, China) at a thickness of 6–8 μm. Lactophenol cotton blue solution was used as mounting medium for examination of anatomic structures and measurements of perithecia, asci and ascospores. Photographs were taken with a Leica DFC450 digital camera (Wetzlar, Germany) attached to a Leica M125 stereomicroscope (Milton Keynes, UK) for gross morphology and a Zeiss AxioCam MRc 5 digital camera (Jena, Germany) attached to a Zeiss Axio Imager A2 microscope (Göttingen, Germany) for microscopic features. Descriptive statistics of ascospores and conidia (minimum, maximum, mean and standard deviation) were calculated following the methods of Hirooka et al. (2012). Measurements of individual structures were based on 30 units, except as otherwise noted. Morphology of colonies were characterised using potato dextrose agar (PDA, 20% w/v potato + 2% w/v dextrose + 2% w/v agar) and synthetic nutrient-poor agar (SNA; Nirenberg 1976) at 25 °C in an incubator with alternating periods of light and darkness (12 h/12 h). Colony growth rates were measured after 7 d.

### DNA extraction, PCR amplification and sequencing

Genomic DNA was extracted from fresh mycelium following the method of Wang and Zhuang (2004). Four primer pairs, act1-act2 (Samuels et al. 2006), ITS5-ITS4 (White et al. 1990), LR0R-LR5 (Vilgalys and Hester 1990; Rehner and Samuels 1994) and crpb1a-rpb1c (Castlebury et al. 2004) were used to amplify the ACT, ITS, LSU and RPB1 regions, respectively. PCR reactions were performed using an ABI 2720 Thermal Cycler (Applied Biosciences, Foster City, USA) with a 25 μl reaction system consisting of 12.5 μl Taq MasterMix, 1 μl each primer (10 μM), 1 μl template DNA and 9.5 μl ddH_2_O, based on the procedures detailed in Chaverri et al. (2011). DNA sequencing was carried out in both directions on an ABI 3730XL DNA Sequencer (Applied Biosciences, Foster City, USA).

### Sequence alignment and phylogenetic analyses

Newly obtained sequences and those retrieved from GenBank are listed in Table 1. The sequences were assembled, aligned and the primer sequences were trimmed via BioEdit 7.0.5 (Hall 1999) and converted to NEXUS files by ClustalX 1.8 (Thompson et al. 1997). A partition homogeneity test was performed with 1,000 replicates in PAUP*4.0b10 (Swofford 2002) to evaluate statistical congruence amongst the four loci. The aligned ACT, ITS, LSU and RPB1 sequences were combined in BioEdit and analysed with Bayesian Inference (BI), Maximum Parsimony (MP) and Maximum Likelihood (ML) methods to determine the phylogenetic positions of the new species. The MP analysis was performed with PAUP 4.0b10 (Swofford 2002) using 1000 replicates of heuristic search with random addition of sequences and subsequent TBR (tree bisection and reconnection) branch swapping. Topological confidence of the resulting trees was tested by Maximum Parsimony bootstrap proportion (MPBP) with 1000 replications, each with 10 replicates of random addition of taxa. The BI analysis was conducted by MrBayes 3.1.2 (Ronquist and Huelsenbeck 2003) using a Markov chain Monte Carlo algorithm. Nucleotide substitution models were determined by MrModeltest 2.3 (Nylander 2004). Four Markov chains were run simultaneously for 1000000 generations with the trees sampled every 100 generations. A 50% majority rule consensus tree was computed after excluding the first 2500 trees as ‘burn-in’. Bayesian Inference posterior probability (BIPP) was determined from the remaining trees. ML analysis was conducted with IQ-Tree 1.6.10 (Nguyen et al. 2015) using the best model for each locus chose by ModelFinder (Chernomor et al. 2016). Branch support measures were calculated with 1000 bootstrap replicates. Trees were examined by TreeView 1.6.6 (Page 1996). *Cosmosporacoccinea* Rabenh. and *Nectriacinnabarina* (Tode) Fr. were used as outgroup taxa. Maximum Likelihood bootstrap proportion (MLBP) and MPBP greater than 50% and BIPP greater than 90% were shown at the nodes.

**Table 1.** List of species, herbarium/strain numbers and GenBank accession numbers of materials used in this study.

**Species**	**Herbarium/strain numbers**	**GenBank Accession numbers**			
		**ACT**	**ITS**	**LSU**	**RPB1**
*Cosmosporacoccinea* Rabenh.	CBS 114050	GQ505967	FJ474072	GQ505990	GQ506020
*Nectriacinnabarina* (Tode) Fr.	AR 4302/AR 4477	HM484627	HM484548	HM484562	HM484577
*Rugonectriacastaneicola* (W. Yamam. & Oyasu) Hirooka & P. Chaverri	CBS 128360	–	MH864901	MH876352	–
*R.microconidia* Z.Q. Zeng & W.Y. Zhuang	HMAS 254521	**MF669044** ^a^	**MF669050**	**MF669052**	**MF669056**
*R.neobalansae* (Samuels) P. Chaverri & Samuels	CBS 125120	–	KM231750	HM364322	KM232146
*R.rugulosa* (Pat. & Gaillard) Samuels, P. Chaverri & C. Salgado	YH 1001	JF832515	JF832661	JF832761	JF832836
*R.sinica* W.Y. Zhuang, Z.Q. Zeng & W.H. Ho	HMAS 183542	**MF669046**	HM054141	HM042430	**MF669058**
*Thelonectriaasiatica* C. Salgado & Hirooka	MAFF 241576	KC121436	KC153774	KC121500	KC153967
*T.beijingensis* Z.Q. Zeng, J. Luo & W.Y. Zhuang	HMAS 188498	**MF669047**	JQ836656	**MF669054**	**MF669059**
*T.blattea* C. Salgado & P. Chaverri	CBS 95268	KC121387	KC153725	KC121451	KC153918
*T.brayfordii* C. Salgado & Samuels	CBS 118612	KC121381	KC153719	KC121445	KC153912
*T.conchyliata* C. Salgado & P. Chaverri	GJS 8745	KC121401	KC153739	KC121465	KC153932
*T.discophora* (Mont.) P. Chaverri & C. Salgado	AR 4742	KC121376	KC153714	KC121440	KC153907
*T.guangdongensis* Z.Q. Zeng & W.Y. Zhuang	HMAS 254522	**MF669045**	**MF669051**	**MF669053**	**MF669057**
*T.ianthina* C. Salgado & Guu	GJS 10118	KC121393	KC153731	KC121457	KC153924
*T.japonica* C. Salgado & Hirooka	MAFF 241524	KC121428	KC153766	KC121492	KC153959
	HMAS 98327	**MK556799**	HM054140	HM042434	–
*T.mammoidea* (W. Phillips & Plowr.) C. Salgado & R.M. Sanchez	IMI 69361	KC121425	KC153763	KC121489	KC153956
*T.ostrina* C. Salgado & P. Chaverri	GJS 9623	KC121418	KC153756	KC121482	KC153949
*T.phoenicea* C. Salgado & P. Chaverri	GJS 85179	KC121398	KC153736	KC121462	KC153929
	HMAS 76856	**MK556800**	JQ836657	DQ119572	–
*T.pinea* (Dingley) C. Salgado & P. Chaverri	AR 4324	HM352875	HM364294	HM364307	HM364326
*T.porphyria* C. Salgado & Hirooka	MAFF 241515	KC121426	KC153764	KC121490	KC153957
	HMAS 98333	**MK556798**	HM054136	HM042433	–
*T.purpurea* C. Salgado & P. Chaverri	GJS 10131	KC121394	KC153732	KC121458	KC153925
*T.rubi* (Osterw.) C. Salgado & P. Chaverri	CBS 11312	KC121380	KC153718	KC121444	KC153911
*T.sinensis* (J. Luo & W.Y. Zhuang) Z.Q. Zeng & W.Y. Zhuang	HMAS 183186	**MF669048**	FJ560441	FJ560436	**MF669060**
*T.tyrus* C. Salgado & P. Chaverri	GJS 9046	KC121413	KC153751	KC121477	KC153944
*T.violaria* C. Salgado & R.M. Sanchez	AR 4766	KC121377	KC153715	KC121441	KC153908
*T.yunnanica* Z.Q. Zeng & W.Y. Zhuang	HMAS 183564	**MF669049**	FJ560438	**MF669055**	**MF669061**

^a^ The GenBank numbers in bold type were newly generated in this study.

## Results

**Figure 1.**
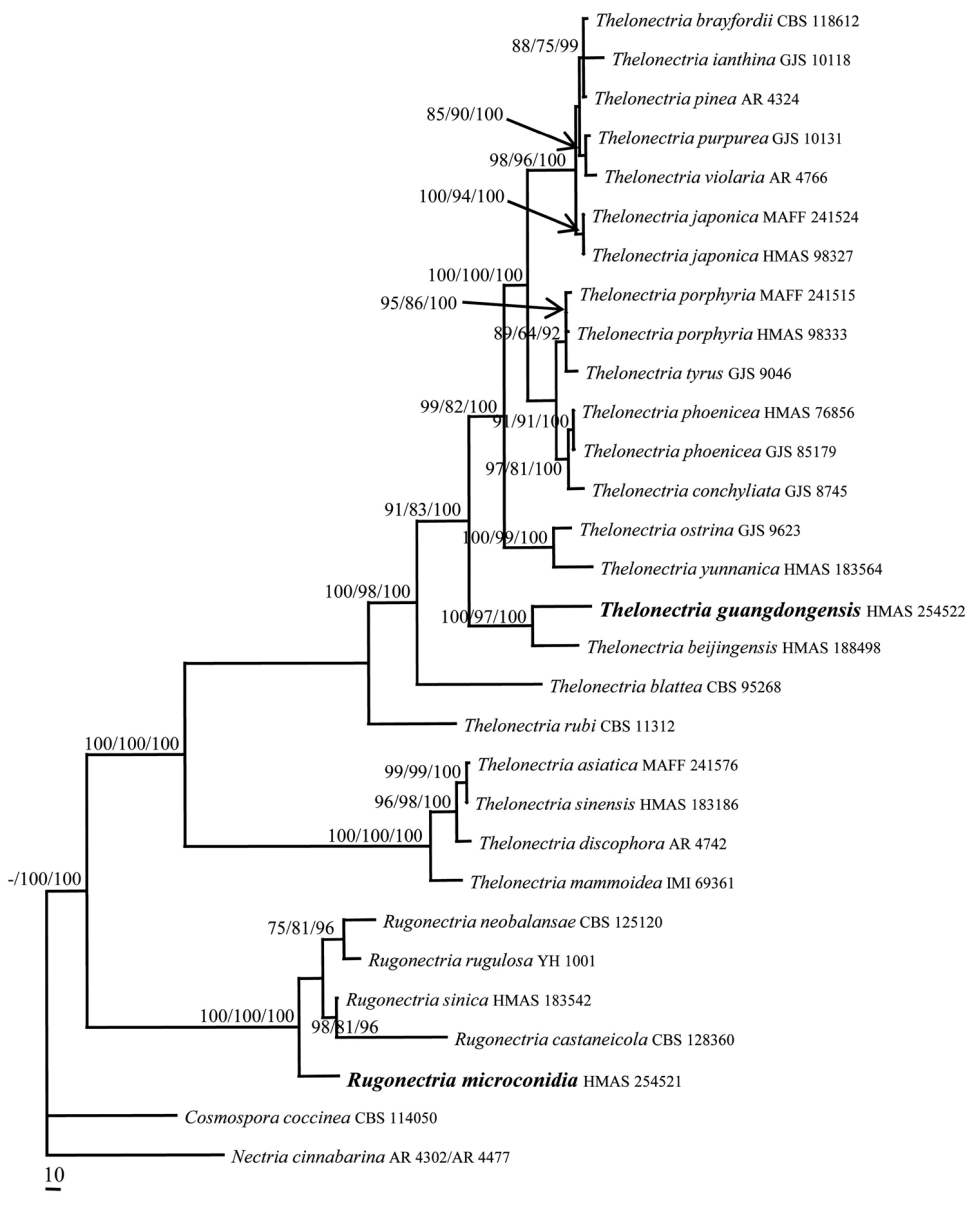
A Maximum Parsimony tree inferred from the combined ACT, ITS, LSU and RPB1 sequences. *Cosmosporacoccinea* and *Nectriacinnabarina* were used as outgroup taxa. MLBP (left) and MPBP (middle) above 50%, BIPP (right) above 90% are indicated at nodes.

The sequences of ACT, ITS, LSU and RPB1 from 25 representative taxa of *Rugonectria* and *Thelonectria* were analysed. The partition homogeneity test (P = 0.03) indicated that the individual partitions were not highly incongruent (Cunningham 1997), thus these four loci were combined for the phylogenetic analyses. In the MP analysis, the datasets included 2524 nucleotide characters, of which 1836 were constant, 198 were variable and parsimony-uninformative and 490 were parsimony-informative. The MP analysis resulted in three most parsimonious trees (tree length = 1415, CI = 0.6721, HI = 0.3279, RI = 0.6098, RCI = 0.5351). One of them is shown in Figure 1. The ML and BI trees were of similar topology. The final matrix was deposited in TreeBASE with accession no. S23994. The isolate HMAS 254521 grouped with other members of *Rugonectria* by receiving high bootstrap values (MLBP/MPBP/BIPP = 100%/100%/100%) and the isolate HMAS 254522 clustered with the representatives of *Thelonectria* (MLBP/MPBP/BIPP = 100%/100%/100%), which support the taxonomic placements of these new species.

### Taxonomy

#### Rugonectria microconidia

Taxon classificationFungiHypocrealesNectriaceae

Z.Q. Zeng & W.Y. Zhuang sp. nov.

Figure 2

##### Holotype.

CHINA. Hunan, Yizhang, Mangshan, (24°57'56.58"N, 112°57'34.63"E), alt. 700 m, on mossy bark, 26 October 2015, Z.Q. Zeng, X.C. Wang, K. Chen, Y.B. Zhang 10266 (HMAS 254521); ex-type culture: HMAS 247232.

##### Sequences.

ACT (MF669044), ITS (MF669050), LSU (MF669052) and RPB1 (MF669056).

##### Etymology.

The specific epithet refers to the microconidia produced in culture.

##### Description.

Mycelium not visible around ascomata or on natural substrata. Ascomata superficial, gregarious, with basal stroma, pyriform to subglobose, non-papillate, yellow to orange, often with a darker red ostiolar area when dry, turning dark red in KOH, becoming slightly yellow in LA, 421–549 × 333–470 μm (n = 8). Perithecial surface warted, 30–93 µm thick, of textura globulosa to textura angularis, cells 10–27 × 8–18 µm, walls 1.5–2.5 µm thick. Perithecial wall of two layers, 45–70 µm thick, outer layer 25–45 µm thick, of textura globulosa to textura angularis; inner layer 7–25 µm thick, of textura prismatica. Asci unitunicate, clavate, 8-spored, 93–130 × (11–)15–25 µm (112.6 ± 12.6 × 18.9 ± 3.2 μm). Ascospores ellipsoid to broadly ellipsoid, 1-septate, striate, uniseriate or biseriate above and uniseriate below, hyaline, 20–28 × 8–12 µm (24.0 ± 2.0 × 10.1 ± 0.9 μm). Colony on PDA 42 mm diameter after 7 d under daylight at 25 °C, surface velvety, with white aerial mycelium, producing pale pinkish pigment in medium. Colony on SNA reaches 40 mm diameter after 7 d under daylight at 25 °C, surface with sparse whitish aerial mycelium. Conidiophores simply branched, 18–50 × 2–3 μm. Microconidia allantoid to rod shaped, slightly curved, 0(1–2)-septate, 3–14(–18) × 1.2–2.5(–3) μm (6.7 ± 3.1 × 1.6 ± 0.4 μm).

**Figure 2.**
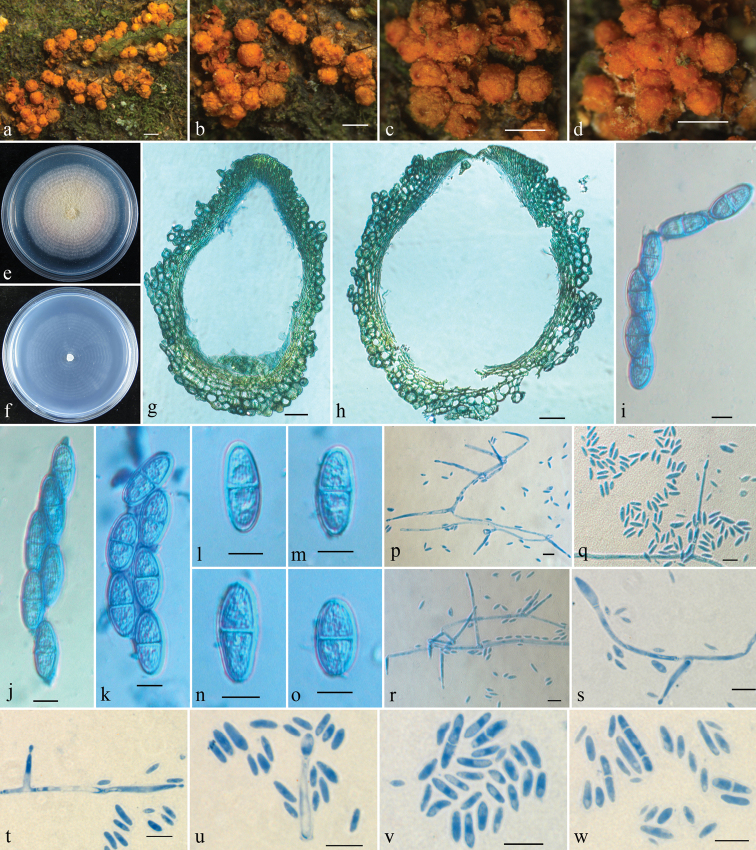
*Rugonectriamicroconidia***a–d** ascomata on natural substratum **e** colony on PDA**f** colony on SNA**g, h** median section through perithecium **i–k** asci with ascospores **l–o** ascospores **p–s** conidiophores and conidia **t, u** conidiogenous cells and conidia **v, w** microconidia. Scale bars: 0.5 mm (**a–d**); 50 μm (**g, h**); 10 μm (**i–w**).

##### Habitat.

On mossy bark.

##### Distribution.

Asia (China).

##### Notes.

The non-papillate perithecia with warted surface, clavate asci with ellipsoidal to broadly ellipsoidal, uniseptate, striate ascospores, as well as our molecular data, suggest that this species belongs to *Rugonectria* (Chaverri et al. 2011). Amongst the known species of the genus, *R.microconidia* is morphologically most similar to the type species, *R.rugulosa*, in having gregarious, warted, orange perithecia often with a dark red ostiole when dry (Samuels et al. 1990; Samuels and Brayford 1994). The newly described species differs in having asci that are 93–130 × (11–)15–25 µm and larger than those of *R.rugulosa* that are (53–)64–83(–95) × (7.5–)11.3–15.5(–17) µm. In addition, the ascospores of *R.microconidia* are also larger, 20–28 × 8–12 µm, while those of *R.rugulosa* are (10–)13.5–18(–24) × (3.3–)4.7–6.7(–10) µm. Unlike *R.microconidia*, *R.rugulosa* does not produce macroconidia in culture (Samuels et al. 1990; Samuels and Brayford 1994). Sequence comparisons reveal that there are 21 bp, 21 bp, 12 bp and 22 bp divergences in the ACT, ITS, LSU and RPB1 regions, respectively, between *R.microconidia* and *R.rugulosa* (YH1001). Both morphological and molecular data suggest that these species are distinct.

#### Rugonectria rugulosa

Taxon classificationFungiHypocrealesNectriaceae

(Pat. & Gaillard) Samuels, P. Chaverri & C. Salgado, in Chaverri, Salgado, Hirooka, Rossman & Samuels, Stud. Mycol. 68: 73, 2011

≡ Nectriarugulosa Pat. & Gaillard, Bull. Soc. Mycol. Fr. 5(4): 115, 1890.
≡ Neonectriarugulosa (Pat. & Gaillard) Mantiri & Samuels, in Mantiri, Samuels, Rahe & Honda, Can. J. Bot. 79(3): 339, 2001.
= Cylindrocarponrugulosum Brayford & Samuels, in Samuels & Brayford, Sydowia 46(1): 148, 1994.

##### Specimens examined.

CHINA. Henan, Jigongshan, alt. 400 m, on rotten twigs, 14 November 2003, W.Y. Zhuang, Y. Nong 5142 (HMAS 91774). Hainan, Changjiang, Bawangling, alt. 1100 m, on rotten twigs, 7 December 2000, W.Y. Zhuang, X.M. Zhang H25 (HMAS 83349); Ledong, Jianfengling, alt. 1100 m, on rotten twigs, 9 December 2000, W.Y. Zhuang, X.M. Zhang, Z.H. Yu H36, H41 (HMAS 83350, 83370); Qiongzhong, Limushan, alt. 700 m, on rotten twigs, 18 December 2000, W.Y. Zhuang, X.M. Zhang H124 (HMAS 76867); Tongzha, Wuzhishan, alt. 1000 m, on bark, 16 December 2000, W.Y. Zhuang, X.M. Zhang, Z.H. Yu, Y.H. Zhang H105 (HMAS 83371); on rotten twigs, W.P. Wu W7058 (HMAS 183161); Yunnan, Xichou, on rotten twigs, 11 November 1999, W.Y. Zhuang, Z.H. Yu 3407 (HMAS 183160).

##### Habitat.

On rotten twigs, wood of recently dead and dying trees.

##### Distribution.

Africa (Congo), Americas (Venezuela), Asia (China, Indonesia), possibly pantropical.

##### Notes.

The species was formerly placed in *Nectria* (Fr.) Fr. and *Neonectria* Wollenw. until Chaverri et al. (2011) introduced *Rugonectria* with *R.rugosa* as the type species. The Chinese materials match well the description of the fungus (Samuels and Brayford 1994).

#### Rugonectria sinica

Taxon classificationFungiHypocrealesNectriaceae

W.Y. Zhuang, Z.Q. Zeng & W.H. Ho, in Zeng, Zhuang & Ho, Mycosystema 31(4): 467, 2013

##### Specimens examined.

CHINA. Hainan, Changjiang, Bawanling, alt. 1100 m, on dead twigs of *Quercus* sp., 7 December 2000, W.Y. Zhuang, X.M. Zhang H22, H30 (HMAS 76854, 83369); Changjiang, Bawanling, alt. 1100 m, on dead twigs, 7 December 2000, W.Y. Zhuang, X.M. Zhang H28 (HMAS 76865); Lingshui, Diaoluoshan, alt. 1100 m, on bark, 13 December 2000, W.Y. Zhuang, X.M. Zhang, Z.H. Yu H70 (HMAS 76866); Henan, Jigongshan, alt. 400 m, on dead twigs, 14 November 2003, W.Y. Zhuang, Y. Nong 5099 (HMAS 91773); Fujian, Wuyishan, on dead twigs, 21 September 2006, W.Y. Zhuang, J. Luo, W.Y. Li 6846 (HMAS 183542).

##### Sequences.

ACT (MF669046), ITS (HM054141), LSU (HM042430) and RPB1 (MF669058).

##### Habitat.

On bark and dead twigs.

##### Distribution.

Asia (China).

##### Notes.

Morphologically *Rugonectriasinica* resembles *R.castaneicola* (W. Yamam. & Oyasu) Hirooka & P. Chaverri in having four-spored asci (Zeng et al. 2012). However, *R.castaneicola* differs in possessing perithecia that are 250–470 × 350–430 μm and larger than those of *R.sinica* that are 216–420 × 194–404 μm. In addition, the ascospores of *R.castaneicola* are larger, 18–28 × 7.5–11 μm, while those of *R.sinica* are 16–26 × 5.5–11 μm. The sequence analyses of the ITS and β-tubulin regions from type culture confirmed that they are different taxa (Zeng et al. 2012).

#### Thelonectria guangdongensis

Taxon classificationFungiHypocrealesNectriaceae

Z.Q. Zeng & W.Y. Zhuang sp. nov.

Figure 3

##### Holotype.

CHINA. Guangdong, Shixing, Chebaling, (24°43'17.38"N, 114°16'39.50"E), alt. 600 m, on branches, 2 November 2015, Z.Q. Zeng, X.C. Wang, K. Chen, Y.B. Zhang 10627 (HMAS 254522); ex-type culture: HMAS 247233.

##### Sequences.

ACT (MF669045), ITS (MF669051), LSU (MF669053) and RPB1 (MF669057).

##### Etymology.

The specific epithet refers to the type locality of the fungus.

##### Description.

Mycelium not visible around ascomata or on natural substrata. Ascomata perithecial, solitary to gregarious, up to 10 in a group, with a well–developed stroma, superficial, subglobose to globose, bright red with a prominently darkened papilla, turning dark red in KOH, becoming slightly yellow in LA, 235–382 × 245–412 μm (n = 8). Perithecial surface slightly roughened. Perithecial wall of two layers, 20–50 µm thick, outer layer 13–37 µm thick, of textura intricata; inner layer 7.5–13 µm thick, of textura prismatica. Asci not observed. Ascospores ellipsoid, 1-septate, smooth, 10–13 × 3–5 µm (11.6 ± 1.3 × 4.2 ± 0.7 μm). Colony on PDA 28 mm diameter after 7 d under daylight at 25 °C, surface velvety, with white aerial mycelium, producing purple pigment in medium. Colony on SNA 35 mm diameter after 7 d under daylight at 25 °C, surface with sparse whitish aerial mycelium. Phialides cylindrical or slightly swollen, 20–58 × 2–4 μm. Macroconidia cylindrical, slightly curved with rounded ends, 2–5-septate, 48–70 × 4.8–5.3 μm (58.9 ± 7.14 × 5.0 ± 0.2 μm). Microconidia and chlamydospores not observed in culture.

**Figure 3.**
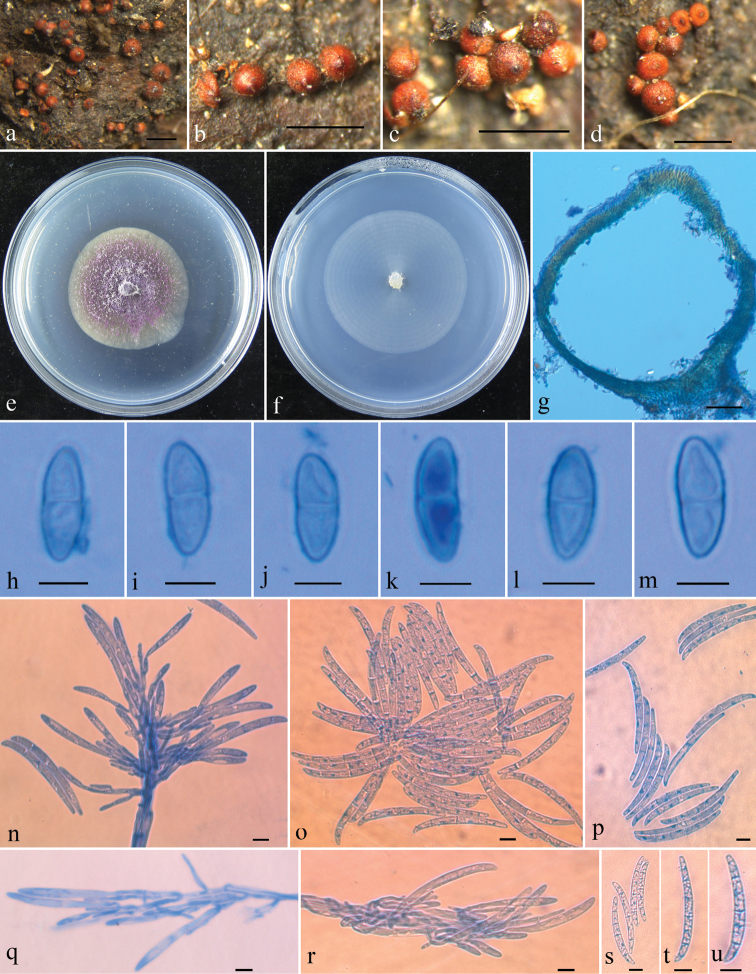
*Thelonectriaguangdongensis***a–d** ascomata on natural substratum **e** colony on PDA**f** colony on SNA**g** median section through perithecium **h–m** ascospores **n, q, r** conidiogenous cells and macroconidia **o, p, s–u** macroconidia. Scale bars: 0.5 mm (**a–d**); 50 μm (**g**); 10 μm (**h–u**).

##### Habitat.

On branches.

##### Distribution.

Asia (China).

##### Notes.

Amongst species of *Thelonectria*, *T.guangdongensis* resembles *T.phoenicea* in having subglobose to globose perithecia with slightly roughened surface, purple colony, lack of microconidia and number of septa in macroconidia (Salgado-Salazar et al. 2015). However, *T.phoenicea* has much larger perithecia 300–600 × 200–350 μm, wider ascospores that are 4–5.5 μm wide, and wider phialides 3–6.5 μm wide (Salgado-Salazar et al. 2015). Moreover, there are 13 bp, 44 bp, 8 bp and 54 bp divergences in the ACT, ITS, LSU and RPB1 regions, respectively, between the type of *T.guangdongensis* (HMAS 254522) and that of *T.phoenicea* (G.J.S. 85–179).

Phylogenetically *T.guangdongensis* is closely related to *T.beijingensis* with strong statistical support (MLBP/MPBP/BIPP = 100%/97%/100%) (Figure 1). However, *T.beijingensis* differs in having larger ascospores that are 13–17 × 4–7 μm, while those of *T.guangdongensis* are 10–13 × 3–5 µm and form microconidia in culture in addition to macroconidia (Zeng and Zhuang 2013). There are 20 bp, 30 bp, 5 bp and 50 bp divergences in the ACT, ITS, LSU and RPB1 regions between the ex-type culture of *T.guangdongensis* and that of *T.beijingensis* (HMAS 188498). Both morphology and molecular data support the establishment of the new species.

#### Thelonectria beijingensis

Taxon classificationFungiHypocrealesNectriaceae

Z.Q. Zeng, J. Luo & W.Y. Zhuang, Phytotaxa 85(1): 18, 2013

##### Specimen examined.

CHINA. Beijing, on bark of an unidentified tree, 1 September 2010, L. Cai 7604 (HMAS 188498), ex-type culture: HMAS 188566.

##### Sequences.

ACT (MF669047), ITS (JQ836656), LSU (MF669054) and RPB1 (MF669059).

##### Habitat.

On bark.

##### Distribution.

Asia (China).

##### Notes.

This species was introduced by Zeng and Zhuang (2013) and only known from the type locality. The phylogenetic analyses indicate that the species is associated with *T.guangdongensis* (Figure 1).

#### Thelonectria coronalis

Taxon classificationFungiHypocrealesNectriaceae

C. Salgado & Guu, in Salgado-Salazar, Rossman, Samuels, Capdet & Chaverri, Mycologia 104(6): 1339, 2012

##### Habitat.

On bark of decaying shrubs and trees.

##### Distribution.

Asia (China).

##### Notes.

Salgado-Salazar et al. (2012) described *T.coronalis*, based on the specimens occurring on bark of decaying shrubs and trees. The fungus is only known from Taipei and Yilan of Taiwan Province.

#### Thelonectria coronata

Taxon classificationFungiHypocrealesNectriaceae

(Penz. & Sacc.) P. Chaverri & C. Salgado, in Chaverri, Salgado, Hirooka, Rossman & Samuels, Stud. Mycol. 68: 76, 2011

≡ Nectriacoronata Penz. & Sacc., Malpighia 11(11–12): 510, 1897.

##### Specimen examined.

CHINA. Hainan, Lingshui, Diaoluoshan, alt. 1050 m, on rotten twigs of *Pinus* sp., 15 December 2000, W.Y. Zhuang, X.M. Zhang H90 (HMAS 76855).

##### Habitat.

On bark of shrubs and trees, sometimes associated with small cankers.

##### Distribution.

Americas (Costa Rica), Asia (Indonesia, Taiwan), possibly pantropical.

##### Notes.

The morphology and molecular data indicated that *T.coronata* is a species complex. Salgado-Salazar et al. (2012) divided it into five taxa on the basis of multigene phylogeny. The Chinese collection matches well the concept of *T.coronata* sensu stricto by Salgado-Salazar et al. (2012).

#### Thelonectria discophora

Taxon classificationFungiHypocrealesNectriaceae

(Mont.) P. Chaverri & C. Salgado, in Chaverri, Salgado, Hirooka, Rossman & Samuels, Stud. Mycol. 68: 76, 2011

≡ Sphaeriadiscophora Mont., Annls Sci. Nat., Bot., sér. 2 3: 353, 1835.
≡ Neonectriadiscophora (Mont.) Mantiri & Samuels, in Mantiri, Samuels, Rahe & Honda, Can. J. Bot. 79(3): 339, 2001.

##### Specimens examined.

CHINA. Hainan, Changjiang, Bawangling, alt. 1100 m, 7 December 2000, on rotten twigs, W.Y. Zhuang, X.M. Zhang, Z.H. Yu H24 (HMAS 83351); Lingshui, Diaoluoshan, alt. 1050 m, 15 December 2000, on rotten twigs, W.Y. Zhuang, X.M. Zhang H83, H92-1 (HMAS 83353, 83352). Yunnan, Tengchong, 16 October 2003, W.P. Wu W7097 (HMAS 183180).

##### Habitat.

On decaying bark of shrubs and trees.

##### Distribution.

Americas (Chile), Asia (China), Europe (Scotland).

**Notes.***Thelonectriadiscophora* is the type species of the genus *Thelonectria*. Many specimens identified as this species were determined to be species complex until Salgado-Salazar et al. (2015) separated them into at least 16 taxa, based on phylogenetic analyses of six nuclear loci and morphological evidences.

#### Thelonectria ianthina

Taxon classificationFungiHypocrealesNectriaceae

C. Salgado & Guu, in Salgado-Salazar, Rossman, Samuels, Hirooka, Sanchez & Chaverri, Fungal Diversity 70(1): 12, 2015

##### Habitat.

On decaying bark of trees and shrubs.

##### Distribution.

Americas (Costa Rica), Asia (China).

##### Notes.

This species is known from Heredia Province of Costa Rica and Taiwan Province of China on decaying bark of trees and shrubs (Salgado-Salazar et al. 2015).

#### Thelonectria japonica

Taxon classificationFungiHypocrealesNectriaceae

C. Salgado & Hirooka, in Salgado-Salazar, Rossman, Samuels, Hirooka, Sanchez & Chaverri, Fungal Diversity 70(1): 14, 2015

##### Specimens examined.

CHINA. Hubei, Wufeng, Houhe, alt. 800 m, 13 September 2004, on rotten twigs, W.Y. Zhuang, Y. Nong 5621 (HMAS 98327); Yunnan, Tengchong, on rotten twigs, W.P. Wu W7104a (HMAS 183155).

##### Sequences.

ACT (MK556799), ITS (HM054140) and LSU (HM042434).

##### Habitat.

On decaying bark of *Faguscrenata* and possibly on bark of other shrubs and trees.

##### Distribution.

Asia (China, Japan).

##### Notes.

Specimens of this fungus were treated as *T.discophora* sensu lato until *T.japonica* was introduced by Salgado-Salazar et al. (2015). The morphological characteristics of the Chinese materials fit the concept of *T.japonica*. The Hubei and Yunnan collections extend its distribution to China.

#### Thelonectria lucida

Taxon classificationFungiHypocrealesNectriaceae

(Höhn.) P. Chaverri & C. Salgado, in Chaverri, Salgado, Hirooka, Rossman & Samuels, Stud. Mycol. 68: 76, 2011

≡ Nectrialucida Höhn., Sber. Akad. Wiss. Wien, Math.-naturw. Kl., Abt. 1 118: 298, 1909.
≡ Neonectrialucida (Höhn.) Samuels & Brayford, in Brayford, Honda, Mantiri & Samuels, Mycologia 96(3): 590, 2004.

##### Habitat.

On decaying bark of shrubs and trees.

##### Distribution.

Africa (Cameroon), Americas (Costa Rica), Asia (China, Indonesia), possibly pantropical.

##### Notes.

This is a relatively common species and recorded as *Neonectrialucida* by Guu et al. (2007) from Taiwan Province.

#### Thelonectria mamma

Taxon classificationFungiHypocrealesNectriaceae

C. Salgado & P. Chaverri, in Salgado-Salazar, Rossman & Chaverri, Fungal Diversity 80: 444, 2016

##### Habitat.

On decaying bark of shrubs and trees.

##### Distribution.

Americas (French Guiana), Asia (China).

##### Notes.

The specimens of this species were filed under *T.lucida* (Guu et al. 2007). After re-examinations of the collections from China and French Guiana, Salgado-Salazar et al. (2016) stated that they represent a separate species related to *T.discophora* sensu stricto.

#### Thelonectria phoenicea

Taxon classificationFungiHypocrealesNectriaceae

C. Salgado & P. Chaverri, in Salgado-Salazar, Rossman, Samuels, Hirooka, Sanchez & Chaverri, Fungal Diversity 70(1): 16, 2015

##### Specimen examined.

CHINA. Hainan, Lingshui, Diaoluoshan, alt. 1050 m, 15 December 2000, W.Y. Zhuang, X.M. Zhang H86 (HMAS 76856).

##### Sequences.

ACT (MK556800), ITS (JQ836657) and LSU (DQ119572).

##### Habitat.

On decaying *Acaciacelsa* and other plants.

##### Distribution.

Asia (China, Indonesia), Oceania (Australia).

##### Notes.

Re-examination of HMAS 76856 indicated that *T.phoenicea* is the correct name for the specimen which was previously identified as *T.discophora*. It is distributed also in Taiwan Province (Salgado-Salazar et al. 2015).

#### Thelonectria porphyria

Taxon classificationFungiHypocrealesNectriaceae

C. Salgado & Hirooka, in Salgado-Salazar, Rossman, Samuels, Hirooka, Sanchez & Chaverri, Fungal Diversity 70(1): 19, 2015

##### Specimen examined.

CHINA. Hubei, Wufeng, Houhe, alt. 800 m, on rotten twigs, 12 September 2004, W.Y. Zhuang, Y. Nong 5542 (HMAS 98333).

##### Sequences.

ACT (MK556798), ITS (HM054136) and LSU (HM042433).

##### Habitat.

On decaying bark of *Cryptomeriajaponica* and other woody substrates.

##### Distribution.

Asia (China, Japan).

##### Notes.

The collection was previously treated as *T.discophora* sensu lato (Zhuang 2013). The sequence analyses (Figure 1) and morphological characteristics of HMAS 98333 indicate that the correct name for the collection is *T.porphyria*.

#### Thelonectria sinensis

Taxon classificationFungiHypocrealesNectriaceae

(J. Luo & W.Y. Zhuang) Z.Q. Zeng & W.Y. Zhuang, Phytotaxa 85(1): 18, 2013

≡ Neonectriasinensis J. Luo & W.Y. Zhuang, Mycologia 102(1): 147, 2010.

##### Specimen examined.

CHINA. Hubei, Shennongjia, alt. 1700 m, on bark of a coniferous (?) tree, 17 September 2003, X.M. Zhang, Y.Z. Wang Z108 (HMAS 183186), ex-type culture: HMAS 173255.

##### Sequences.

ACT (MF669048), ITS (FJ560441), LSU (FJ560436) and RPB1 (MF669060).

##### Habitat.

On bark of a coniferous (?) tree.

##### Distribution.

Asia (China).

##### Notes.

The species was originally placed in *Neonectria* by Luo and Zhuang (2010). The anatomic structures and DNA data support its placement in *Thelonectria* (Zeng and Zhuang 2013).

#### Thelonectria veuillotiana

Taxon classificationFungiHypocrealesNectriaceae

(Sacc. & Roum.) P. Chaverri & C. Salgado, Stud. Mycol. 68: 77, 2011

≡ Nectriaveuillotiana Sacc. & Roum., Rev. Mycol. 2: 189, 1880.
≡ Neonectriaveuillotiana (Sacc. & Roum.) Mantiri & Samuels, Canda. J. Bot. 79: 339, 2001.

##### Specimens examined.

CHINA. Anhui, Jinzhai, Tiantangzhai, alt. 1000 m, on bark, 24 August 2011, W.Y. Zhuang, H.D. Zheng, Z.Q. Zeng, S.L. Chen 7869 (HMAS 266577). Hubei, Shennongjia, alt. 1200 m, on rotten twigs associated with other fungi, 15 September 2004, W.Y. Zhuang, Y. Nong 5686 (HMAS 98332); Shennongjia, alt. 1700 m, on bark associated with other fungi, 15 September 2003, X.M. Zhang, Y. Z. Wang Z196 (HMAS 183188); Xingshan, Longmenhe, alt. 1800 m, on rotten twigs associated with other fungi, 18 September 2004, W.Y. Zhuang, Y. Nong 5832 (HMAS 99207). Jilin, Changbaishan, alt. 800 m, on rotten twigs, 27 July 2012, T. Bau, W.Y. Zhuang, H.D. Zheng, Z.Q. Zeng, Z.X. Zhu, F. Ren 8246 (HMAS 266579); Jiaohe, Qianjin forest farm, alt. 450 m, on rotten twigs, 23 July 2012, T. Bau, W.Y. Zhuang, Z.Q. Zeng, H.D. Zheng, Z.X. Zhu, F. Ren 8087b (HMAS 266578). Yunnan, Tengchong, on rotten twigs associated with other fungi, 16 September 2003, W.P. Wu W7095 (HMAS 183568).

##### Sequences.

ITS (HM054151) and LSU (HM042437).

##### Habitat.

On bark of deciduous trees, *Eucalyptus* sp., *Fagus* sp., *Gleditschiatriacanthos*, *Salix* sp.

##### Distribution.

Asia (China), Europe (France and Germany), Azores Islands.

##### Notes.

The species was first placed in *Nectria*, then in *Neonectria* (Mantiri et al. 2001) and recently transferred to *Thelonectria* by Chaverri et al. (2011). It occurs on bark of recently killed trees, rarely on wood or leaves and is cosmopolitan in distribution (Brayford and Samuels 1993; Zhuang 2013).

#### Thelonectria yunnanica

Taxon classificationFungiHypocrealesNectriaceae

Z.Q. Zeng & W.Y. Zhuang, Phytotaxa 85(1): 19, 2013

##### Specimen examined.

CHINA. Yunnan, Baoshan, on bark of an unidentified tree, 15 October 2003, W.P. Wu W7122 (HMAS 183564), ex-type culture: HMAS 188567.

##### Sequences.

ACT (MF669049), ITS (FJ560438), LSU (MF669055) and RPB1 (MF669061).

##### Habitat.

On bark.

##### Distribution.

Asia (China).

##### Notes.

*Thelonectriayunnanica* is only known from the type locality. It is phylogenetically related to *T.ostrina* (Figure 1). However, *T.ostrina* has a perithecial wall 25–40 μm while those of *T.yunnanica* are thicker 49–71 μm and have asci that are (56–)67–86(−98) × 7–12 μm while those of *T.yunnanica* are larger, 87–120 × 8.2–9.6 μm. Unlike *T.yunnanica*, *T.ostrina* does not forming microconidia in culture (Zeng and Zhuang 2013; Salgado-Salazar et al. 2015).

### Excluded species

#### Thelonectria jungneri

Taxon classificationFungiHypocrealesNectriaceae

(Henn.) P. Chaverri & C. Salgado, in Chaverri, Salgado, Hirooka, Rossman & Samuels, Stud. Mycol. 68: 76, 2011

≡ Nectriajungneri Henn., Bot. Jb. 22: 75, 1895.
≡ Neonectriajungneri (Henn.) Samuels & Brayford, Mycologia 96(3): 580, 2004.
≡ Macronectriajungneri (Henn.) C. Salgado & P. Chaverri, in Salgado-Salazar, Rossman & Chaverri, Fungal Diversity 80: 448, 2016.

##### Specimen examined.

CHINA. Guangdong, Dinghushan, on rotten twigs associated with other fungi, 9 October 1998, W.P. Wu W1871-2 (HMAS 183155).

##### Habitat.

On various woody substrates, as well as other plant organic matter.

##### Distribution.

Africa (Cameroon), Americas (Brazil, Costa Rica), Asia (China), possibly pantropical.

##### Notes.

This fungus was originally described as *Nectriajungneri* and was transferred to *Neonectria* (Brayford et al. 2004) and *Thelonectria* (Chaverri et al. 2011). The recent work by Salgado-Salazar et al. (2016) indicated that it belongs to a separate genus *Macronectria* C. Salgado & P. Chaverri.

## Discussion

The genus *Rugonectria* is characterised by the non-papillate, orange to red, conspicuously warted to rugose perithecial surface (Chaverri et al. 2011). The ascomatal anatomy, perithecial wall reactions to KOH and LA, features of asci and ascospores and asexual states indicate the placement of *R.microconidia* in this genus. The multi-locus sequence analyses confirm our morphological observations (Figure 1) and it is here described as a new species.

Historically, the nectriaceous fungi with cylindrocarpon-like asexual states were assigned to *Neonectria*. The accumulated morphological and phylogenetic data suggest that the genus was heterogeneous (Mantiri et al. 2001). Efforts were made towards establishment of a monophyletic *Neonectria* as well as its allies (Booth 1966, Rossman et al. 1999; Mantiri et al. 2001; Brayford et al. 2004). The previously recognised infrageneric groups within *Neonectria* are now recognised as separate genera, i.e. *Ilyonectria* for the *N.radicicola*-group, *Neonectria* sensu stricto for the *N.coccinea*-group, *Rugonectria* for the *N.rugulosa*-group and *Thelonectria* for the *N.mammoidea/N.veuillotiana*-groups (Chaverri et al. 2011). Since the establishment of *Thelonectria*, 45 species have been placed in the genus (www.indexfungorum.org). Salgado-Salazar et al. (2012, 2015) suggested that the criteria formerly used for generic differentiation were of insufficient sensitivity to accurately reflect the degree of species diversity within the group. Subsequently, Salgado-Salazar et al. (2016) emended the generic concept of *Thelonectria* by excluding *T.jungneri*, based on the molecular data and morphological characteristics.

The type species of *Thelonectria*, *T.discophora*, previously considered to be cosmopolitan, was first described based on material collected from Chile and was determined to be heterogeneous (Brayford et al. 2004). Salgado-Salazar et al. (2015) provided a revisionary treatment of the *T.discophora* species complex and recognised 16 cryptic species on the basis of the combined analyses of phylogeny and morphology. In this study, the new species *T.guangdongensis* is determined to be congeneric with *T.discophora*, while both the molecular data and morphological characteristics indicate that *T.guangdongenis* is distinct from other species of *Thelonectria*. To date, 11 species of *Thelonectria* have been recorded from China (Teng 1936; Salgado-Salazar et al. 2012, 2015, 2016; Zeng and Zhuang 2012; Zhuang 2013). China is extremely diverse in its climate, vegetation, geographic structures and multiple niches. Our understanding of species diversity of the nectriaceous fungi will be significantly broadened in the near future.

## Supplementary Material

XML Treatment for Rugonectria microconidia

XML Treatment for Rugonectria rugulosa

XML Treatment for Rugonectria sinica

XML Treatment for Thelonectria guangdongensis

XML Treatment for Thelonectria beijingensis

XML Treatment for Thelonectria coronalis

XML Treatment for Thelonectria coronata

XML Treatment for Thelonectria discophora

XML Treatment for Thelonectria ianthina

XML Treatment for Thelonectria japonica

XML Treatment for Thelonectria lucida

XML Treatment for Thelonectria mamma

XML Treatment for Thelonectria phoenicea

XML Treatment for Thelonectria porphyria

XML Treatment for Thelonectria sinensis

XML Treatment for Thelonectria veuillotiana

XML Treatment for Thelonectria yunnanica

XML Treatment for Thelonectria jungneri
